# Shared Enrichment of the *MUC5B* Variant rs35705950 in Idiopathic Pulmonary Fibrosis and Fibrotic Hypersensitivity Pneumonitis: Evidence from a Russian Cohort

**DOI:** 10.3390/diagnostics16203383

**Published:** 2026-10-18

**Authors:** Vladimir O. Sigin, Tamara A. Savina, Nikolay N. Chekanov, Sergey N. Avdeev, Natalya V. Trushenko, Olga A. Suvorova, Baina B. Lavginova, Iuliia A. Levina, Olesya V. Kuryleva, Goulnara Yu. Babadjanova, Viktor M. Misharin

**Affiliations:** 1 Research Centre for Medical Genetics, Moskvorechye St., 1, Moscow 115522, Russia; 2 Pulmonology Scientific Research Institute, Orekhovyy Bul’var 28, Moscow 115682, Russia; 3 Federal Scientific and Clinical Center for Specialized Types of Medical Care and Medical Technologies, Orekhovyy Bul’var 28, Moscow 115682, Russia; 4 “Biotek Campus” Limited Liability Company, Miklukho-Maklaya St. 16/10, Build. 16, Moscow 117437, Russia; 5 Department of Pulmonology, N.V. Sklifosovsky Institute of Clinical Medicine, I.M. Sechenov First Moscow State Medical University ( Sechenov University), Trubetskaya St. 8, Build. 2, Moscow 119991, Russia; 6 Department of Multidisciplinary Clinical Training, Faculty of Fundamental Medicine, M.V. Lomonosov Moscow State University, Leninskie Gory 1, Build. 40, Moscow 119992, Russia

**Keywords:** *MUC5B* rs35705950 polymorphism, idiopathic pulmonary fibrosis, fibrotic hypersensitivity pneumonitis, interstitial lung disease

## Abstract

**Background/Objectives**: The *MUC5B* rs35705950 polymorphism is the strongest common genetic risk factor for idiopathic pulmonary fibrosis (IPF), whereas its role in fibrotic hypersensitivity pneumonitis (fHP) remains incompletely characterized, particularly in Eastern European cohorts. This study aimed to evaluate the distribution of *MUC5B* rs35705950 genotypes and alleles in Russian patients with IPF and fHP and to assess their association with disease susceptibility using a large external Russian reference dataset. **Methods**: The study included 114 patients with interstitial lung disease: 50 with IPF and 64 with fHP. Genotyping was performed using a custom PCR-restriction fragment length polymorphism assay. Genotype and allele frequencies were compared with an external reference dataset from the National Genetic Initiative of Russia (*n* = 89,261). Association analyses were performed under dominant T-carrier and allelic models. **Results**: The rs35705950 T allele was significantly enriched in both IPF and fHP compared with the external reference dataset. T-carrier genotypes were detected in 52.0% of patients with IPF and 42.2% of patients with fHP, compared with 18.9% in the reference dataset. T-allele frequencies were 28.0%, 26.6%, and 10.0%, respectively. In the dominant model, T-carrier status was associated with increased odds of IPF (OR = 4.65; 95% CI, 2.56–8.46; *p* < 0.001) and fHP (OR = 3.13; 95% CI, 1.83–5.29; *p* < 0.001). The direct comparison between IPF and fHP was inconclusive because of limited statistical power. **Conclusions**: In this Russian clinical cohort, the rs35705950 T allele was more frequent in patients with IPF and fHP than in the external Russian reference dataset. These findings provide regional replication of previously reported associations. The study did not assess causal mechanisms or diagnostic performance.

## 1. Introduction

Over recent decades, interstitial lung diseases (ILDs) have moved from a relatively narrow specialist domain to a growing focus of clinical and scientific attention [1,2]. This shift reflects their increasing contribution to chronic respiratory morbidity, disability, and mortality worldwide. In the Global Burden of Disease 2023 estimates, absolute disability-adjusted life-years (DALYs) attributable to interstitial lung disease and pulmonary sarcoidosis increased 2.4-fold between 1990 and 2023 [3]. These data underscore the need to reconsider how interstitial lung diseases are approached in primary care, where they remain under-recognized despite their growing clinical relevance. Diagnostic delays of one to three years are still frequently reported in fibrotic ILDs, largely reflecting low disease awareness, non-specific respiratory symptoms, and delayed referral to specialist ILD centers. Recent primary care-based data show that structured ILD recognition pathways combined with direct referral to specialist centers can reduce the time from symptom onset to diagnosis from 22.1 to 6 months, underscoring diagnostic delay as a modifiable barrier to timely specialist assessment and initiation of appropriate therapy [4].

Clinically, ILDs usually have a gradual onset, most often presenting with exertional dyspnea and persistent cough. With disease progression, these symptoms are accompanied by declining exercise tolerance and an increasing symptom burden, particularly fatigue. Less specific constitutional manifestations, including anorexia and unintentional weight loss, may also occur, especially in progressive fibrotic disease and selected ILD subtypes [5,6,7].

Idiopathic pulmonary fibrosis (IPF) is a specific form of chronic, progressive fibrosing interstitial pneumonia of unknown cause that occurs predominantly in older adults, is confined to the lungs, and is characterized by the histopathological and/or radiological pattern of usual interstitial pneumonia (UIP) [8]. Current models of IPF pathogenesis place recurrent injury to a vulnerable alveolar epithelium at the center of disease initiation. Failed epithelial repair and aberrant re-epithelialization create a profibrotic alveolar niche, promoting fibroblast activation, myofibroblast expansion, and excessive extracellular matrix deposition. Over time, this process drives progressive remodeling of the lung parenchyma, with normal alveolar architecture being replaced by fibrotic scar tissue [9,10].

The *MUC5B* variant rs35705950 was first reported by Seibold et al. as a major susceptibility locus for pulmonary fibrosis and has since become the most consistently replicated common genetic risk factor for idiopathic pulmonary fibrosis. Located approximately 3 kb upstream of the *MUC5B* transcription start site, the minor allele is markedly enriched in patients with idiopathic pulmonary fibrosis and familial interstitial pneumonia and is associated with increased *MUC5B* expression in lung tissue [11]. Mechanistically, this association is thought to reflect aberrant *MUC5B* expression in distal airway and alveolar epithelial compartments, where excessive mucin production may impair mucociliary clearance, alter epithelial host defense, and promote maladaptive repair after repetitive epithelial injury. Experimental data support this airway-centered model: *Muc5b* is required for normal mucociliary clearance and airway defense, whereas *Muc5b* overexpression in bronchoalveolar epithelium impairs mucociliary clearance and enhances the extent and persistence of bleomycin-induced lung fibrosis in mice [12]. Together, these findings position *MUC5B* not merely as a genetic susceptibility marker for idiopathic pulmonary fibrosis, but as a biologically plausible contributor to fibrotic remodeling, supporting further investigation of its role across fibrotic interstitial lung diseases.

Hypersensitivity pneumonitis (HP) is an inflammatory and/or fibrotic disease of the lung parenchyma and small airways that develops in susceptible individuals following inhalational exposure to antigens that trigger an immune-mediated response [13]. According to current clinical practice guidelines, hypersensitivity pneumonitis is classified into two phenotypes: nonfibrotic HP (nfHP) and fibrotic HP (fHP), the latter of which is associated with a progressive disease course and poor prognosis. According to the American College of Chest Physicians (CHEST) guidelines, a diagnosis of HP can be established in patients with a documented antigen exposure and typical high-resolution computed tomography (HRCT) features of the disease [14].

Genetic susceptibility is increasingly recognized as an important modifier of hypersensitivity pneumonitis, particularly in its fibrotic form. A partial genetic overlap between fHP and IPF has been reported, with common IPF risk variants in *TERC*, *DSP*, *MUC5B*, and IVD showing significant associations with fHP [15]. Among these loci, the *MUC5B* variant rs35705950 is of particular interest: in chronic hypersensitivity pneumonitis, the minor allele has been associated with disease risk and greater radiographic extent of fibrosis, supporting the concept that *MUC5B*-related epithelial and mucociliary dysfunction may contribute to fibrotic remodeling beyond IPF [16]. Recent data further suggest that *TOLLIP* and *MUC5B* polymorphisms and haplotypes may influence susceptibility and survival in fHP, highlighting these variants as candidate markers of disease phenotype and prognosis [17].

However, rs35705950 frequencies vary substantially across geographic regions, and data on its clinical relevance in fHP and IPF remain unevenly represented across regional cohorts [18,19,20]. This gap is particularly relevant for Russia, where the *MUC5B* variant has not been extensively characterized in clinically defined ILD cohorts in comparison with a large regional reference dataset.

In the present study, we characterized the genotype distribution and allele frequencies of the *MUC5B* variant rs35705950, with particular focus on the risk-associated T allele, in a clinically defined Russian cohort of patients with fibrotic hypersensitivity pneumonitis and idiopathic pulmonary fibrosis, using a large external Russian reference dataset for comparison.

Our group previously reported an initial Russian-language analysis of rs35705950 in 37 patients with IPF and 49 patients with HP using the same external reference dataset [21]. All 86 patients from that report were included in the present cohort. All 49 patients in the earlier HP group had fibrotic HP; no non-fibrotic HP cases were included in that group. The present study extends this clinical series to 50 patients with IPF and 64 with fHP and provides Sanger confirmation of representative genotypes, genotype–phenotype analysis, a more rigorous statistical evaluation incorporating multiple-testing correction and power assessment, and a dedicated cross-cohort comparison of rs35705950 frequencies across geographic and ancestry-defined groups.

## 2. Materials and Methods

### 2.1. Patients and Reference Dataset

A total of 114 patients from Russia with confirmed interstitial lung disease were included in the study. Based on the clinical diagnosis, patients were assigned to two groups: fibrotic hypersensitivity pneumonitis (fHP; *n* = 64) and idiopathic pulmonary fibrosis (IPF; *n* = 50).

Diagnoses of IPF and fHP were established through multidisciplinary discussion involving experienced pulmonologists and thoracic radiologists, with pathologists participating when histopathological material was available.

The diagnosis of fHP was established according to the 2020 ATS/JRS/ALAT clinical practice guideline [13] through integrated assessment of clinical manifestations, occupational and environmental exposure history, serum antigen-specific IgG results when available, HRCT findings, bronchoalveolar lavage (BAL) fluid analysis, and histopathological findings when available. BAL lymphocytosis greater than 20% was considered a finding supporting the diagnosis of HP. HRCT patterns were categorized as typical of, compatible with, or indeterminate for fibrotic HP. A typical fibrotic HP pattern was characterized by radiological evidence of pulmonary fibrosis, including reticular abnormalities, traction bronchiectasis, or honeycombing, together with at least one feature of small airway disease, such as ill-defined centrilobular nodules, mosaic attenuation, or air trapping on expiratory images. Surgical lung biopsy was performed in selected diagnostically indeterminate cases when clinically appropriate.

IPF was diagnosed according to the 2022 ATS/ERS/JRS/ALAT clinical practice guideline [8] after exclusion of known causes of interstitial lung disease, including relevant occupational or environmental exposure, connective tissue disease, and drug toxicity. The diagnosis was based primarily on a UIP or probable UIP pattern on HRCT and, when available, the corresponding histopathological findings. A UIP pattern was characterized by subpleural and basal-predominant fibrosis with honeycombing, with or without peripheral traction bronchiectasis or bronchiolectasis, and without HRCT features suggesting an alternative diagnosis.

In patients with a UIP-like fibrotic pattern, differentiation between fHP and IPF was based on multidisciplinary integration of exposure history, features of small airway disease on HRCT, BAL lymphocytosis, and histopathological findings when available; the imaging pattern was not interpreted in isolation.

Allele and genotype frequencies were compared with an external Russian reference dataset from the National Genetic Initiative of Russia (*n* = 89,261). Aggregate genotype and allele frequencies for rs35705950 were provided by a study coauthor through a collaboration with Biotek Campus (organization website: https://www.biotechcampus.ru/, access on 6 September 2026). Approximately 50% of the samples were obtained from generally healthy adult volunteers recruited in urban centers across Russia, whereas the remaining samples were provided by partner medical organizations. Related individuals were not excluded. The supplied frequencies were not stratified by age or sex, and age and sex distributions and diagnostic information were not provided to the authors. Individual-level reference data were not available to the authors. The dataset was not assembled as an individually matched disease-free control cohort and was therefore used as an external reference for allele and genotype frequencies.

The study was conducted in accordance with the principles of the Declaration of Helsinki and was approved by the Local Ethics Committee of Federal State Budgetary Institution “Pulmonology Scientific Research Institute” under the Federal Medical and Biological Agency of the Russian Federation (protocol No. 04/24, dated 24 June 2024). Written informed consent was obtained from all patients included in the clinical cohort.

### 2.2. DNA Extraction

Genomic DNA was isolated from peripheral blood samples using the standard phenol–chloroform extraction method. DNA concentration was measured fluorometrically using a (Thermo Fisher Scientific, Waltham, MA, USA) with the Qubit dsDNA Assay Kit according to the manufacturer’s instructions. DNA purity was assessed spectrophotometrically using a Nano-500 microvolume UV–Vis spectrophotometer (Hangzhou Allsheng Instruments Co., Ltd., Hangzhou, China) based on the A260/A280 and A260/A230 absorbance ratios. DNA samples of sufficient concentration and purity were subsequently used for molecular genetic analysis.

### 2.3. MUC5B rs35705950 Genotyping

Genotyping of the *MUC5B* single-nucleotide polymorphism rs35705950 was performed using a custom-designed PCR-RFLP assay. The amplified fragment contained the polymorphic restriction site affected by rs35705950 and an additional non-polymorphic site that served as an internal control of restriction enzyme activity.

### 2.4. Polymerase Chain Reaction

To amplify the target *MUC5B* locus (GRCh38: chr11:1,219,802–1,220,144), the following primers were used: forward, 5′-CCTGGCCAGAATGAGGGACAGT-3′; reverse, 5′-GGCAGAGGGCCCATTGGGA-3′. The amplified fragment was 343 bp in length. PCR was performed in a final volume of 25 µL containing 1× ammonium sulfate PCR buffer (67 mM Tris-HCl, pH 8.8 at 25 °C; 16.6 mM (NH_4_)_2_SO_4_; 0.01% Tween-20), 2 mM MgCl_2_, 0.2 mM of each dNTP, 0.5 µM of each primer, 1 U of Taq DNA polymerase, and 50 ng of genomic DNA. The amplification conditions were as follows: initial denaturation at 95 °C for 5 min, followed by 32 cycles of denaturation at 95 °C for 10 s, annealing at 64 °C for 20 s, and extension at 72 °C for 20 s, with a final extension at 72 °C for 10 min. The annealing temperature of 64 °C was optimized empirically.

### 2.5. Restriction Digestion of PCR Products

Restriction digestion was performed using BstHHI restriction endonuclease (SibEnzyme Ltd., Novosibirsk, Russia), which recognizes the GCG↑C/C↓GCG site. The 20 µL reaction mixture, containing 10 µL of PCR product, 1× Buffer Y, and 1 U of restriction enzyme, was incubated overnight at 50 °C for approximately 16 h. Restriction products were visualized by polyacrylamide gel electrophoresis (PAGE), followed by silver nitrate staining.

All 114 patient samples yielded interpretable genotype calls, corresponding to a call rate of 100%. No samples required repeat genotyping. Genotype assignment was accepted only when the expected fragment generated by the non-polymorphic BstHHI site was present, thereby providing an internal control of restriction enzyme activity. No-template controls were included to monitor PCR contamination. Representative samples corresponding to the GG, GT, and TT restriction patterns were independently analyzed by Sanger sequencing, and complete concordance between PCR-RFLP and Sanger sequencing was observed.

Complete gel documentation for all 114 patient samples and a summary of genotyping quality-control metrics are provided in Figure S1 and Table S4, respectively.

### 2.6. Statistical Analysis

Continuous variables were summarized as mean ± standard deviation or median with interquartile range, depending on distributional properties and reporting convention. Categorical variables were summarized as counts and percentages.

Hardy–Weinberg equilibrium was assessed separately in the fHP group, the IPF group, and the external reference dataset using an exact test. Pairwise comparisons of genotype distributions between cohorts were performed using 2 × 3 contingency tables including GG, GT, and TT genotypes. Fisher–Freeman–Halton exact tests were used where appropriate. For large contingency tables involving the reference dataset, Monte Carlo simulation with 1,000,000 replicates was applied when exact computation was impractical.

Allele-frequency comparisons were performed using 2 × 2 contingency tables for T and G alleles.

The association between rs35705950 and disease status was evaluated using odds ratios with 95% confidence intervals. Two models were analyzed: a dominant T-carrier model, defined as GT + TT versus GG, and an allelic model, defined as T versus G. Patient groups were compared with the external reference dataset described above. Because individual-level age, sex, and ancestry covariates were unavailable for this dataset, covariate-adjusted logistic regression was not performed.

Genotype–phenotype analyses evaluated associations between rs35705950 T-carrier status and the 10 demographic and clinical variables reported in Table 1. Because TT homozygotes were infrequent, analyses used a dominant model (GT + TT versus GG) in the combined patient cohort and separately in the IPF and fHP groups. Continuous variables were compared using two-sided Mann–Whitney U tests and categorical variables using two-sided Fisher exact tests; available-case analysis was used for each variable.

The larger numbers of available percent-predicted than absolute values (FVC: 111 versus 103; DLCO: 110 versus 101) reflected incomplete capture of absolute values during clinical data collection. Available percent-predicted values were retained, and missing absolute values did not exclude patients from analyses of other available variables.

To account for multiple pairwise testing, all *p* values requiring correction were adjusted using the Holm method. Correction was performed within the corresponding families of analyses, including genotype-distribution comparisons, allele-frequency comparisons, association tests for the dominant T-carrier and allelic models, and genotype–phenotype analyses. Odds ratios and 95% confidence intervals were reported as unadjusted effect-size estimates, whereas the corresponding *p* values were corrected for multiple testing where applicable.

The clinical sample size was determined by the number of clinically characterized patient samples available for inclusion; therefore, no formal a priori sample-size calculation was performed. An approximate post hoc power analysis based on Cohen’s h for two independent proportions was performed using the observed T-carrier frequencies and a two-sided α of 0.05. The estimated power was 99.9% for the comparison of IPF with the external reference dataset, 98.4% for fHP with the external reference dataset, and 18.1% for the direct comparison between IPF and fHP. Accordingly, the latter comparison was not used to infer equivalence or similarity between the clinical groups.

All tests were two-sided, and corrected *p* values below 0.05 were considered statistically significant.

All analyses were performed in R version 4.4.2; graphical summaries were generated using ggplot2 version 4.0.0.

## 3. Results

The final study cohort comprised 114 patients with confirmed interstitial lung disease, including 64 patients with fHP and 50 patients with IPF. The cohort included 58 men and 56 women. The median age was 64 (56–70) years in the fHP group and 68 (63–75) years in the IPF group. For comparative analysis, genotype and allele frequencies were assessed against a reference dataset from the National Genetic Initiative of Russia (*n* = 89,261). The baseline clinical characteristics of the study cohort are summarized in Table 1.

In the fHP group, an inciting antigen was identified in 39 of 64 patients (60.9%). The HRCT pattern was classified as typical of fibrotic HP in all 64 patients. A UIP-like pattern was documented in 18 patients (28.1%), was absent in 39 (60.9%), and its status was unavailable for the remaining seven (10.9%). All 39 patients with an identified antigen belonged to the non-UIP subgroup. BAL was performed in 10 patients (15.6%); BAL lymphocytosis, defined as a lymphocyte proportion greater than 20%, was present in four of these 10 patients (40%). Histopathological assessment was available in seven patients (10.9%). The association of the rs35705950 T allele with fHP persisted in a sensitivity analysis restricted to patients with an identified antigen and a non-UIP pattern (Supplementary Table S5).

### 3.1. RFLP Assay for MUC5B rs35705950 Genotyping

A 343 bp amplicon spanning the *MUC5B* rs35705950 locus (GRCh38: chr11:1,219,802–1,220,144) was subjected to digestion with BstHHI. The polymorphic BstHHI recognition site encompassing rs35705950 was located at chr11:1,219,989–1,219,992, whereas the additional non-polymorphic BstHHI site at chr11:1,219,908–1,219,911 served as an internal digestion control. Within the amplicon, these sites corresponded to nucleotides 188–191 and 107–110, respectively. This design enabled reliable discrimination between true heterozygous samples and incomplete digestion of homozygous samples.

**Figure 1:**
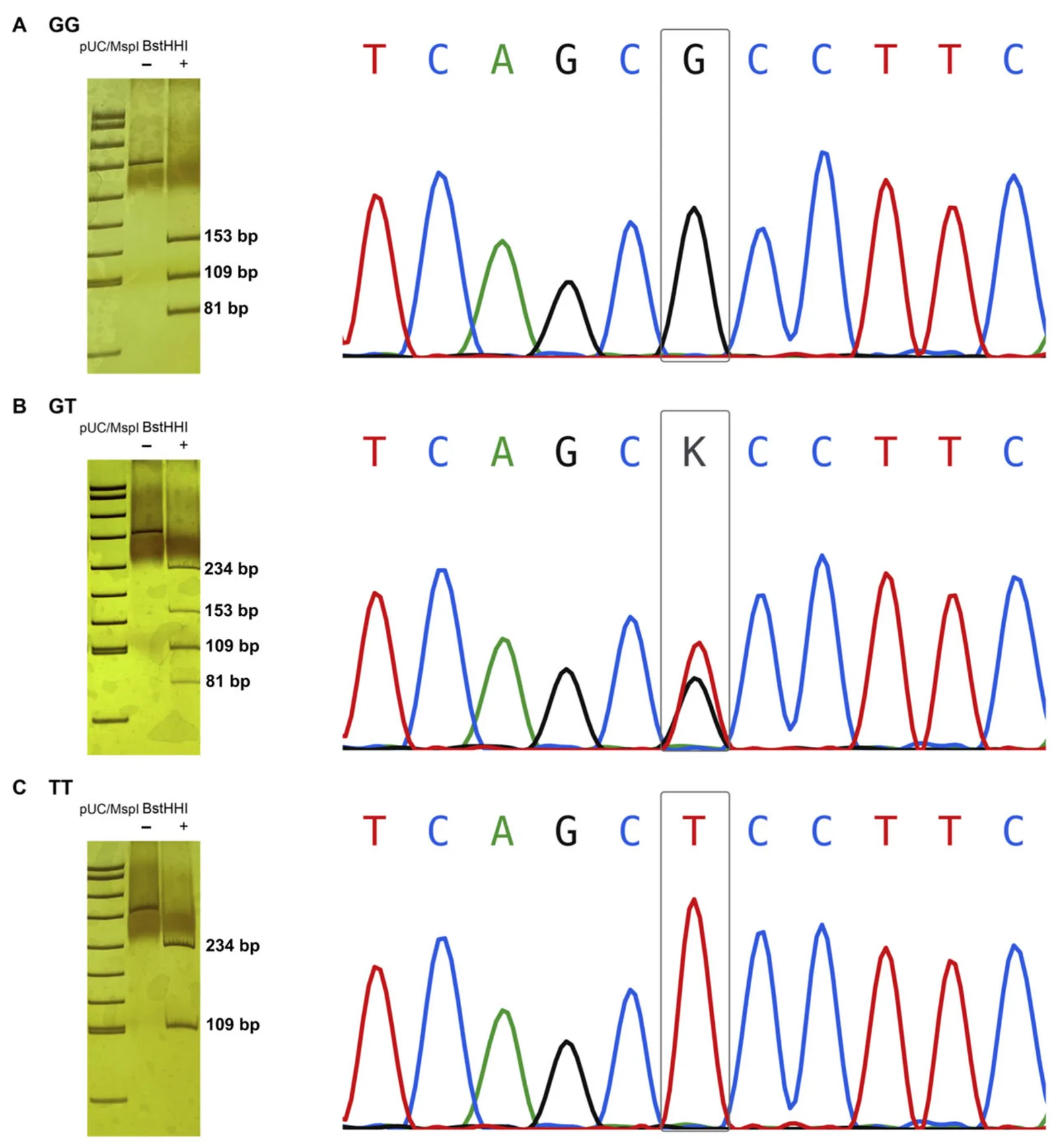
Representative PCR-RFLP patterns and Sanger confirmation of *MUC5B* rs35705950 genotypes. Representative undigested (−) and BstHHI-digested (+) PCR products are shown for the GG (**A**), GT (**B**), and TT (**C**) genotypes, together with Sanger sequencing chromatograms from one representative sample of each genotype. Expected restriction fragment sizes are labelled beside the bands: GG, 153, 109, and 81 bp; GT, 234, 153, 109, and 81 bp; TT, 234 and 109 bp. The 109 bp fragment serves as an internal control of restriction enzyme activity. The rs35705950 position is boxed; K denotes the heterozygous G/T call. pUC19/MspI was used as the DNA size marker. In the Sanger sequencing chromatograms, nucleotide labels and corresponding peaks are color-coded as follows: A (green), T (red), G (black), and C (blue).

The expected fragment patterns after BstHHI digestion were as follows: GG, 153, 109, and 81 bp; GT, 234, 153, 109, and 81 bp; and TT, 234 and 109 bp (Figure 1). The presence of the 109 bp fragment generated by cleavage at the non-polymorphic site confirmed successful enzymatic digestion, whereas genotype assignment was based on the allele-specific 234 bp or 153- and 81 bp fragments.

### 3.2. Genotype and Allele Frequencies of MUC5B rs35705950

**Figure 2:**
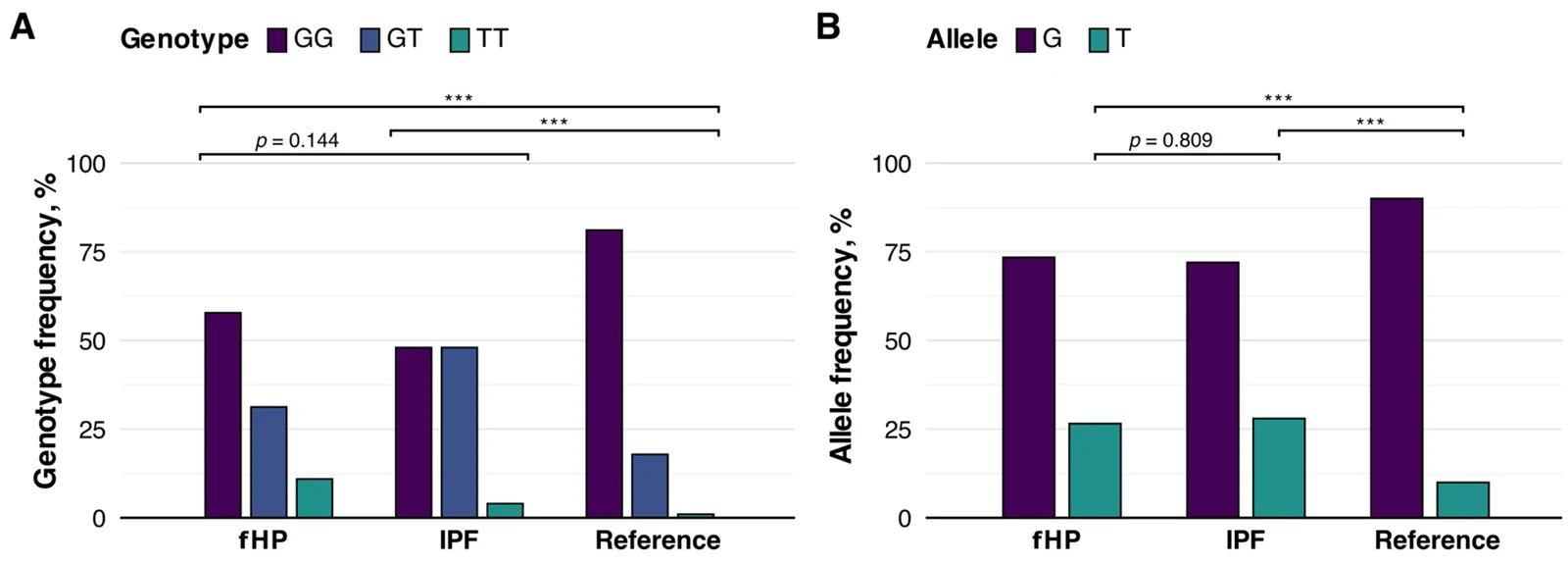
Genotype distribution and allele frequencies of the *MUC5B* rs35705950 polymorphism in patients with fibrotic hypersensitivity pneumonitis, idiopathic pulmonary fibrosis, and in the reference dataset. The left panel (**A**) shows the distribution of GG, GT, and TT genotypes across the study groups. The right panel (**B**) shows the corresponding frequencies of the G and T alleles. Brackets indicate pairwise comparisons of genotype distributions or allele frequencies; *p* values were adjusted using the Holm method, and *** indicates *p* < 0.001.

Genotyping of the *MUC5B* rs35705950 polymorphism was performed in patients with fHP and IPF, and the results were compared with the reference dataset. Genotype and allele distributions are presented in Table 2 and Figure 2.

The genotype distribution in the reference dataset was consistent with Hardy–Weinberg equilibrium (*p* = 0.380). No significant deviation from Hardy–Weinberg equilibrium was observed in either patient group: fHP, *p* = 0.115; IPF, *p* = 0.294. Compared with the reference dataset, the rs35705950 T allele was markedly enriched in both clinical groups. T-carrier genotypes were detected in 26 of 50 patients with IPF (52%) and 27 of 64 patients with fHP (42.2%), compared with 16,871 of 89,261 individuals in the reference dataset (18.9%). This shift was reflected by a substantially lower proportion of GG homozygotes in fHP and IPF than in the reference dataset (57.8% and 48% vs. 81.10%, respectively), together with higher frequencies of the GT genotype (31.2% and 48% vs. 17.88%) and the TT genotype (10.9% and 4% vs. 1.02%). Consistently, the T allele frequency was almost threefold higher in IPF and 2.7-fold higher in fHP than in the reference dataset (28% and 26.56% vs. 9.96%, respectively).

Pairwise analysis confirmed significant differences between each patient group and the reference dataset for both genotype distribution and allele frequency (*p* < 0.001). In the direct comparison between IPF and fHP, no statistically significant difference was observed in genotype distribution (*p* = 0.144) or T-allele frequency (*p* = 0.809). Under the dominant model, T-carrier status was numerically more frequent in IPF than in fHP (52.0% vs. 42.2%; OR = 1.48; 95% CI, 0.66–3.34; *p* = 0.346). The wide confidence interval precludes conclusions regarding either a difference or similarity between the clinical groups.

### 3.3. Association of MUC5B rs35705950 with fHP and IPF

**Figure 3:**
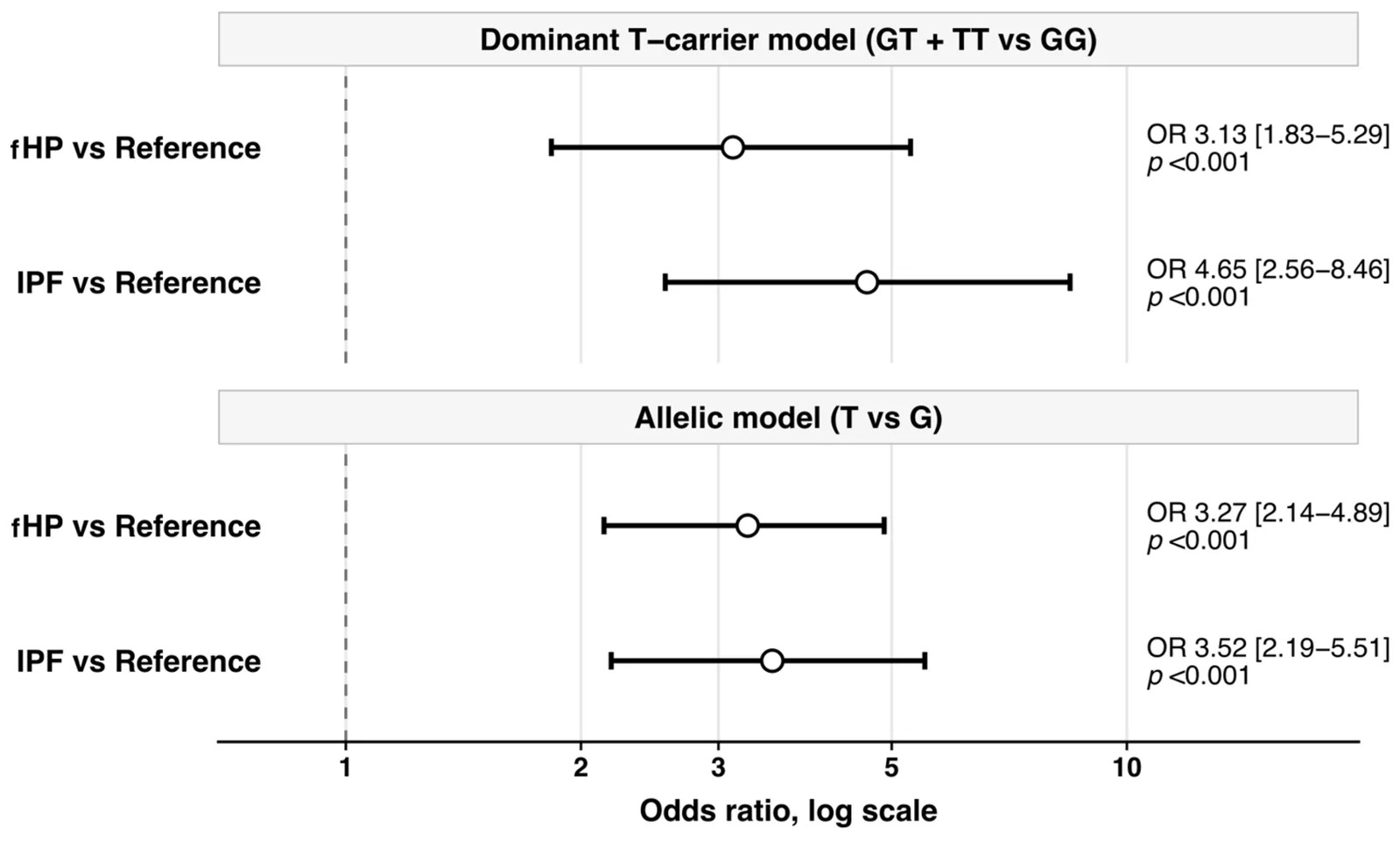
Association of *MUC5B* rs35705950 with fibrotic hypersensitivity pneumonitis and idiopathic pulmonary fibrosis. Odds ratios were calculated relative to the reference dataset under two genetic models: the dominant T-carrier model (GT + TT vs. GG) and the allelic model (T vs. G). Points indicate odds ratios, and horizontal bars indicate 95% confidence intervals. The dashed vertical line represents OR = 1.

Association analysis was performed to quantify the relationship between *MUC5B* rs35705950 and disease status using odds ratios calculated relative to the reference dataset (Figure 3).

In the dominant T-carrier model (GT + TT vs. GG), carriage of the rs35705950 T allele was associated with increased odds of both fHP and IPF. The effect estimate was numerically higher for IPF (OR = 4.65; 95% CI, 2.56–8.46; *p* < 0.001), while a significant association was also observed for fHP (OR = 3.13; 95% CI, 1.83–5.29; *p* < 0.001).

A consistent pattern was observed in the allelic model (T vs. G). The T allele was associated with increased odds of fHP (OR = 3.27; 95% CI, 2.14–4.89; *p* < 0.001) and IPF (OR = 3.52; 95% CI, 2.19–5.51; *p* < 0.001). Overall, both genetic models supported a significant association of rs35705950 with the two clinical groups relative to the reference dataset.

### 3.4. Genotype–Phenotype Analysis

Genotype–phenotype analyses under the dominant model were performed in the combined patient cohort and separately within the fHP and IPF groups (Tables S1–S3; Figures S2–S4). No association between T-carrier status and any of the 10 assessed clinical characteristics remained significant after Holm adjustment. The lowest unadjusted *p* values were observed for age in the combined cohort (*p* = 0.094; adjusted *p* = 0.935), BMI and age in fHP (*p* = 0.053 and *p* = 0.054, respectively; adjusted *p* = 0.526 for both), and FVC in liters in IPF (*p* = 0.078; adjusted *p* = 0.783).

## 4. Discussion

In this study, we assessed the distribution of the *MUC5B* polymorphism rs35705950 in patients with idiopathic pulmonary fibrosis and fibrotic hypersensitivity pneumonitis relative to genotype and allele frequencies in a large reference dataset. The primary focus was the enrichment of the rs35705950 T allele in fibrotic interstitial lung diseases and its association with disease status. Genotype–phenotype analyses were also performed in the combined patient cohort and separately within the fHP and IPF groups. No association between T-carrier status and the assessed cross-sectional clinical characteristics remained significant after Holm adjustment (Tables S1–S3; Figures S2–S4). The main finding of our study is that the rs35705950 T allele was enriched not only in patients with IPF, but also in those with fHP. Although the dominant-model point estimate was numerically higher for IPF, the direct comparison between the clinical groups was underpowered and does not establish a difference between them. This pattern suggests that rs35705950 should not be viewed solely as a disease-specific marker of IPF, but may also reflect a broader genetic contribution to fibrotic remodeling in interstitial lung disease. However, the present association study does not establish a shared causal mechanism between IPF and fHP. The absence of robust associations between rs35705950 status and clinical or laboratory characteristics in our cohort does not negate the primary genetic association, but indicates that genotype–phenotype effects, if present, are likely to be modest and require larger, clinically homogeneous cohorts for reliable detection.

The 2025 ERS/ATS statement on the international multidisciplinary classification of interstitial pneumonias emphasizes the growing relevance of molecular and genetic predictors, including *MUC5B* rs35705950, in the classification and interpretation of fibrotic interstitial lung diseases. According to this consensus, one copy of the *MUC5B* T allele increases the risk of IPF approximately sixfold, whereas two copies increase the risk approximately twentyfold. Importantly, the consensus also notes that a high prevalence of the *MUC5B* polymorphism may be observed not only in IPF, but also in fHP with a usual interstitial pneumonia pattern and in rheumatoid arthritis-associated interstitial lung disease, suggesting a potential shared genetic basis across these conditions [22].

Functional evidence further supports the relevance of the *MUC5B* locus to IPF susceptibility. Borie et al. [23] used colocalization of expression quantitative trait loci and DNA methylation quantitative trait loci with genetic risk loci to demonstrate an association between rs35705950 and changes in *MUC5B* expression and DNA methylation in lung tissue. These findings provide mechanistic support for a functional role of the *MUC5B* locus in the etiology of IPF.

In line with this, Stock et al. [24] showed that rs35705950 T-allele carriage was associated with increased *MUC5B* protein expression in the distal airways and honeycomb cysts of patients with IPF, whereas no comparable T-allele-dependent difference was observed in idiopathic nonspecific interstitial pneumonia, systemic sclerosis-associated nonspecific interstitial pneumonia, or control lung tissue. These observations suggest that the relationship between rs35705950 and increased *MUC5B* expression may be particularly relevant to the IPF lung microenvironment, rather than representing a uniform mechanism across all interstitial lung diseases.

Recent case–control data have further strengthened the evidence for a genotype-dose relationship between rs35705950 and IPF susceptibility. Mouawad et al. [18] studied 55 patients with IPF and 94 healthy controls and showed that the variant was significantly enriched among patients in both the heterozygous and homozygous states. GT and TT genotypes were detected in 50.9% and 20.0% of IPF cases, respectively, compared with 31.9% and 4.3% of controls, while the minor T-allele frequency was 45.5% in IPF and 20.2% in controls. The magnitude of association increased with the number of T alleles: compared with the GG genotype, the GT genotype was associated with a 3.50-fold increase in IPF odds, whereas the TT genotype was associated with a 10.31-fold increase. These findings support the interpretation of the T allele as a risk-associated variant rather than a marker of background allele frequency alone. At the broader ILD level, the large meta-analysis by Lou et al. [25] showed that the association of rs35705950 is not uniform across all interstitial lung diseases, but is most prominent in idiopathic and familial interstitial pneumonias. In an analysis of 16 publications comprising 28 studies, 76,345 cases, and 18,402 controls, rs35705950 was associated with a significantly increased risk of idiopathic interstitial pneumonia across all genetic models, including a strong effect for the TT genotype compared with GG. Subgroup analyses showed the strongest associations for idiopathic pulmonary fibrosis and idiopathic nonspecific interstitial pneumonia, whereas no significant association was found for systemic sclerosis-associated interstitial lung disease. These data support the view that the rs35705950 T allele is more closely linked to idiopathic and familial forms of interstitial pneumonia than to fibrotic lung involvement across all systemic or secondary ILD contexts.

Evidence from HP studies indicates that the relevance of rs35705950 is not confined to IPF. Ley et al. [16] examined two independent cohorts of patients with chronic hypersensitivity pneumonitis (cHP), including 145 patients from the University of California, San Francisco and 72 from the University of Texas Southwestern Medical Center, and found that the minor allele frequency of rs35705950 was significantly higher in patients with cHP than in controls, reaching levels comparable to those observed in IPF. In the combined cohort, rs35705950 minor alleles were associated with a greater extent of radiographic fibrosis. In parallel, short telomere length was associated with more extensive fibrosis, histopathological features of usual interstitial pneumonia, and reduced survival. These findings point to the potential value of *MUC5B* rs35705950, alongside telomere-related markers, for risk stratification in patients with cHP.

Additional studies have supported the clinical relevance of rs35705950 in fHP. In a case–control analysis including 97 patients with fHP and 112 controls, Mota et al. [17] found that the minor allele frequency of rs35705950 was significantly higher in patients than in controls (40.7% vs. 12.1%). Specific *MUC5B*/*TOLLIP* haplotypes were associated not only with disease susceptibility but also with poorer survival, and the authors also reported associations between *TOLLIP* variants and reduced baseline lung function, as well as between rs35705950 and radiological features of fibrosis. Furusawa et al. [15] provided complementary genetic evidence by showing that several common genetic risk variants previously associated with IPF were also associated with fHP, with the strongest association observed for rs35705950 in *MUC5B*. Together, these findings support the concept that fHP and IPF share elements of genetic susceptibility architecture.

Lewandowska et al. [26] further highlighted the potential clinical significance of rs35705950 in fHP beyond disease susceptibility alone. In a cohort of 86 consecutive patients with fHP, the minor T-allele frequency was 17.4%, with GG observed in 69.8% of patients and GT/TT genotypes in 30.2%. Although the presence of the T allele was not significantly associated with a higher prevalence of lung fibrosis on high-resolution computed tomography, GT/TT carriers had significantly lower baseline forced vital capacity and more frequently demonstrated a decline in FVC over time. Patients carrying the T allele also tended to show a poorer response to immunomodulatory treatment and more often required antifibrotic therapy. These observations suggest that, in fHP, rs35705950 may be related more closely to disease behavior and functional progression than to the mere presence of radiological fibrosis.

The magnitude of the rs35705950 association should be interpreted in the context of ancestry-specific background allele frequencies. In the comparative summary reported by Mouawad et al. [18], the rs35705950 T-allele frequency among patients with IPF varied markedly across cohorts, ranging from very low values in Korean, Chinese/East Asian, and Japanese groups to approximately 27–42% in most European and North American cohorts, with the highest value reported in the Lebanese cohort. Control frequencies were consistently lower than case frequencies, but followed a similar background-dependent pattern.

Notably, the rs35705950 T-allele frequency in the National Genetic Initiative of Russia dataset (9.96%) was close to the frequency reported for European-ancestry controls from the 1000 Genomes Project (10.7%) [16], providing independent support that the reference frequency is consistent with previously reported European population data.

In our Russian cohort, the T-allele frequency in IPF was 28%, with GT and TT genotypes observed in 48% and 4% of patients, respectively, compared with a T-allele frequency of 10.0% in the reference dataset. These values are broadly consistent with the range reported for European and North American cohorts and clearly differ from the much lower frequencies generally reported in East Asian cohorts.

For HP, published data are more limited and less systematically stratified by ancestry, but they similarly suggest enrichment of rs35705950 in chronic or fibrotic disease forms. Reported T-allele frequencies include 17.4% in the Polish HP cohort [26], 24.4–32.3% in US cHP cohorts [16], and 40.7% in the Portuguese fHP cohort [17].

In our Russian fHP cohort, the T-allele frequency was 26.6%, with GT and TT genotypes detected in 31.2% and 10.9% of patients, respectively. These values fall within the range reported for chronic or fibrotic HP, although direct comparisons should be made cautiously because published studies differ in ancestry, diagnostic criteria, fibrotic disease burden, biological material analyzed, and control selection.

Overall, our findings support two complementary conclusions. First, rs35705950 shows reproducible disease-associated enrichment in both IPF and fHP. Second, the absolute T-allele frequency varies markedly by ancestry background, which is critical for interpreting case–control estimates across countries. In this context, the reference frequency observed in our dataset is consistent with European population data, and the T allele is enriched in both IPF and fHP. The direct comparison between IPF and fHP was underpowered, and the wide confidence interval precluded conclusions regarding either a difference or similarity between the groups. Nevertheless, the enrichment of the T allele in each clinical group relative to the reference dataset is consistent with previously reported evidence of partially overlapping genetic susceptibility across fibrotic interstitial lung diseases. Therefore, *MUC5B* rs35705950 should be considered a risk-associated variant contributing to fibrotic susceptibility across ILD phenotypes, rather than a standalone disease-specific diagnostic marker. Its interpretation should be integrated with clinical, radiological, histopathological, and background allele-frequency data.

The main limitations of this study include the relatively small IPF and fHP groups and the limited number of TT homozygotes, which restricted genotype-specific analyses, reduced the power of the genotype–phenotype comparisons, and left the direct IPF–fHP comparison underpowered. The external reference dataset was not individually matched to the patient groups, and individual-level covariates were unavailable for this dataset; therefore, residual ancestry-related confounding cannot be excluded, and the reported odds ratios are unadjusted. Related individuals were not excluded from the reference dataset, so its observations may not all be independent. Diagnostic information was unavailable, and some reference samples originated from medical organizations; the inclusion of individuals with ILD therefore cannot be excluded. Patient and reference genotypes were generated by PCR-RFLP and whole-genome sequencing, respectively; however, internal digestion controls and concordance of representative GG, GT, and TT genotypes with Sanger sequencing supported reliable genotype assignment. Despite guideline-based multidisciplinary diagnosis, residual diagnostic heterogeneity within the fHP group cannot be entirely excluded; treatment, longitudinal progression, and survival were not evaluated, and only a single genetic variant was analyzed without haplotype or polygenic analyses.

## 5. Conclusions

Thus, our study showed that the *MUC5B* rs35705950 T allele is enriched in patients with IPF and fHP compared with a large reference dataset, with a numerically higher dominant-model effect estimate in IPF. Given the variability of rs35705950 frequencies across genetic backgrounds and geographic regions, these findings underscore the need to interpret genetic associations against appropriate reference allele-frequency data. Overall, rs35705950 likely reflects not only susceptibility to IPF but also a broader genetic contribution to fibrotic interstitial lung disease. Larger studies using well-matched control cohorts are needed to clarify its relationship with clinical phenotype, disease progression, and prognosis.

## Figures and Tables

**Table 1:** Clinical and demographic characteristics of the study cohort.

Variable	fHP (*n* = 64)	IPF (*n* = 50)	*p* Value
Age, years	64 (56–70)	68 (63–75)	0.011
Male sex, *n* (%)	20 (31.2)	38 (76.0)	<0.001
BMI, kg/m^2^	29.49 ± 5.33 (28.15–30.84)	28.99 ± 5.05 (27.56–30.43)	0.613
FVC, L	2.25 (1.84–3.01)	2.59 (2.07–3.27)	0.209
FVC, % predicted	70 ± 23 (65–76)	70 ± 19 (64–75)	0.861
DLCO, mL/min/mmHg	8.81 (6.27–11.61)	8.48 (6.17–10.52)	0.394
DLCO, % predicted	43.0 (34.0–62.0)	41.0 (33.5–48.0)	0.120
Smoking, *n* (%)	15 (23.4)	26 (52.0)	0.002
COPD, *n* (%)	1 (1.6)	6 (12.0)	0.043
Hypertension, *n* (%)	47 (74.6)	40 (80.0)	0.498

Normally distributed continuous variables are shown as mean ± SD [95% CI]; non-normally distributed variables are shown as median (IQR). Categorical variables are shown as *n* (%). Normality was assessed using the Shapiro–Wilk test separately within each cohort. Continuous variables were compared using Welch t-test or Mann–Whitney U test, as appropriate. Categorical variables were compared using the Pearson chi-square test or Fisher exact test. Summary statistics and percentages were calculated among non-missing values. Abbreviations: fHP, fibrotic hypersensitivity pneumonitis; IPF, idiopathic pulmonary fibrosis; BMI, body mass index; FVC, forced vital capacity; DLCO, diffusing capacity of the lungs for carbon monoxide; COPD, chronic obstructive pulmonary disease.

**Table 2:** Genotype distribution and allele frequencies of the MUC5B rs35705950 polymorphism in patients with IPF and fHP and in the reference dataset.

Cohort	Total, *n*	TT, *n* (%)	GT, *n* (%)	GG, *n* (%)	T Allele Frequency	G Allele Frequency
fHP	64	7 (10.9)	20 (31.2)	37 (57.8)	0.2656	0.7344
IPF	50	2 (4)	24 (48)	24 (48)	0.2800	0.7200
Reference dataset	89,261	909 (1.02)	15,962 (17.88)	72,390 (81.10)	0.0996	0.9004

## Data Availability

The de-identified individual genotype and phenotype data for the 114 patients are provided in Data S1 of the Supplementary Materials. The aggregated genotype and allele counts for the external reference dataset are reported in Table 2. Individual-level data from the external reference dataset were not available to the authors.

## Supplementary Materials

The following supporting information can be downloaded at: https://www.mdpi.com/article/10.3390/diagnostics16203383/s1. Data S1: de-identified patient-level genotype and phenotype data; Tables S1–S3: genotype–phenotype comparisons; Table S4: PCR–RFLP genotyping quality-control summary; Table S5: sensitivity analysis in patients with fHP who had both an identified inciting antigen and a non-UIP HRCT pattern; Figure S1: complete PCR–RFLP gel documentation; Figures S2–S4: genotype–phenotype analysis figures.

## Abbreviations

The following abbreviations are used in this manuscript:

ALAT	Latin American Thoracic Society
ATS	American Thoracic Society
BAL	Bronchoalveolar lavage
BMI	Body mass index
CI	Confidence interval
COPD	Chronic obstructive pulmonary disease
DALY	Disability-adjusted life-year
DLCO	Diffusing capacity of the lungs for carbon monoxide
ERS	European Respiratory Society
fHP	Fibrotic hypersensitivity pneumonitis
cHP	Chronic hypersensitivity pneumonitis
FVC	Forced vital capacity
HP	Hypersensitivity pneumonitis
HRCT	High-resolution computed tomography
ILD	Interstitial lung disease
IPF	Idiopathic pulmonary fibrosis
IQR	Interquartile range
JRS	Japanese Respiratory Society
OR	Odds ratio
PAGE	Polyacrylamide gel electrophoresis
PCR	Polymerase chain reaction
PCR-RFLP	Polymerase chain reaction–restriction fragment length polymorphism
SD	Standard deviation
SNP	Single-nucleotide polymorphism
UIP	Usual interstitial pneumonia
