# Supplementary material for: Shared Enrichment of the MUC5B Variant rs35705950 in Idiopathic Pulmonary Fibrosis and Fibrotic Hypersensitivity Pneumonitis: Evidence from a Russian Cohort
Source: Diagnostics (Basel). 2026 Oct 18;16(20):3383. doi: 10.3390/diagnostics16203383 (PMC13703225; doi:10.3390/diagnostics16203383)
Supplement: Supplementary file 1 [file diagnostics-16-03383-s001.zip › diagnostics-4580991-supplementary.pdf]

MDPI

diagnostics

*Supplementary Materials*

# Shared Enrichment of the *MUC5B* Variant rs35705950 in Idiopathic Pulmonary Fibrosis and Fibrotic Hypersensitivity Pneumonitis: Evidence from a Russian Cohort

**Vladimir O. Sigin, Tamara A. Savina, Nikolay N. Chekanov, Sergey N. Avdeev, Natalya V. Trushenko, Olga A. Suvorova, Baina B. Lavginova, Iuliia A. Levina, Olesya V. Kuryleva, Goulnara Yu. Babadjanova, and Viktor M. Misharin**

## Supplementary Methods

*Dataset and genotype–phenotype analysis*

The genotype–phenotype analysis included 114 clinically characterized patients, including 64 with fibrotic hypersensitivity pneumonitis (fHP) and 50 with idiopathic pulmonary fibrosis (IPF). The analysis was restricted to the 10 demographic and clinical variables reported in Table 1 of the main manuscript: age, sex, body mass index, forced vital capacity in liters and percent predicted, diffusing capacity for carbon monoxide in absolute units and percent predicted, smoking, chronic obstructive pulmonary disease, and hypertension.

Because TT homozygotes were uncommon, genotype–phenotype comparisons used a dominant model, contrasting GG homozygotes with carriers of the T allele (GT + TT). Continuous variables were summarized as median [Q1–Q3] and compared using the two-sided Mann–Whitney U test. Categorical variables were summarized as n/N (%) for the positive category and compared using the two-sided Fisher exact test. Available-case analysis was used for each variable. Holm adjustment was applied across the 10 variables separately in the combined, fHP, and IPF analyses.

*PCR–RFLP quality control and gel documentation*

All 114 patient samples yielded interpretable PCR–RFLP genotype calls (call rate, 100%); there were no failed calls and no samples required repeat genotyping. The non-polymorphic BstHHI recognition site within the amplicon served as an internal control of restriction enzyme activity. No-template controls were included to monitor PCR contamination. One representative sample of each genotype (GG, GT, and TT) was independently confirmed by Sanger sequencing, with complete concordance (3/3); these chromatograms are presented in Figure 1 of the main manuscript. Figure S1 provides complete gel documentation for all patient lanes.

**Data S1.** Individual genotype and phenotype data. fHP cohort, patients 1-32 of 64.

| Sample | Diagnosis | Genotype | T-carrier group | Age (years) | Sex | BMI ($kg/m^2$) | FVC (L) | FVC (% predicted) | DLCO (mL/min/mmHg) | DLCO (% predicted) | Smoking | COPD | Hypertension |
|---|---|---|---|---|---|---|---|---|---|---|---|---|---|
| HP5 | fHP | GG | GG | 52 | Female | NA | 2.94 | 91 | NA | NA | No | NA | NA |
| HP7 | fHP | TT | GT+TT | 48 | Female | 30.1 | 1.96 | 56 | 11.43 | 50 | No | No | No |
| HP8 | fHP | GT | GT+TT | 72 | Female | 31.6 | 1.51 | 50 | 4.96 | 26 | No | No | Yes |
| HP9 | fHP | GT | GT+TT | 71 | Female | 23.4 | 1.27 | 52 | 6.13 | 36 | No | No | No |
| HP11 | fHP | GG | GG | 24 | Female | 20.1 | 1.43 | 40 | 5.89 | 28 | No | No | No |
| HP12 | fHP | GG | GG | 47 | Female | 24.6 | 3.31 | 29 | 5.75 | 29 | No | No | No |
| HP13 | fHP | GG | GG | 19 | Male | 29.7 | 2.09 | 34 | 4.71 | 34 | No | No | No |
| HP14 | fHP | GG | GG | 62 | Female | 31.6 | 1.18 | 38 | 6.55 | 33 | No | No | Yes |
| HP15 | fHP | GG | GG | 52 | Female | 24.4 | 1.11 | 37 | 6.9 | 31 | No | No | Yes |
| HP16 | fHP | GG | GG | 67 | Female | 35.7 | 3.14 | 116 | 12.41 | 69 | No | No | Yes |
| HP17 | fHP | TT | GT+TT | 70 | Female | 26.5 | 2.15 | 73 | 11.98 | 62 | No | No | No |
| HP18 | fHP | GG | GG | 69 | Male | 31.7 | 4.46 | 92 | 9.62 | 34 | Yes | No | Yes |
| HP19 | fHP | GT | GT+TT | 69 | Female | 35.5 | 5.38 | 57 | 5.01 | 30 | No | No | Yes |
| HP20 | fHP | GT | GT+TT | 78 | Male | 31.2 | 1.81 | 64 | 8.05 | 43 | No | No | Yes |
| HP21 | fHP | GT | GT+TT | 49 | Female | 37.2 | 1.83 | 51 | 8.05 | 39 | No | No | No |
| HP22 | fHP | GG | GG | 65 | Female | 29.3 | 1.49 | 51 | 6.99 | 37 | No | No | Yes |
| HP23 | fHP | GT | GT+TT | 61 | Female | 19.9 | 2 | 63 | 5.71 | 29 | No | No | Yes |
| HP24 | fHP | GT | GT+TT | 63 | Male | 28 | 3.58 | 89 | 9.6 | 65 | No | No | Yes |
| HP26 | fHP | GG | GG | 74 | Female | 28 | 2.43 | 109 | 10.8 | 67 | No | No | Yes |
| HP27 | fHP | GG | GG | 68 | Female | 30.8 | 2.76 | 76 | 8.81 | 39 | Yes | No | Yes |
| HP28 | fHP | GG | GG | 68 | Female | 31 | 2.43 | 85 | 2.34 | 67 | No | No | Yes |
| HP29 | fHP | TT | GT+TT | 71 | Female | 36.5 | 2.65 | 111 | 2.25 | 68 | No | No | Yes |
| HP30 | fHP | GG | GG | 57 | Male | 33.1 | 3.83 | 63 | 2.56 | 64 | No | No | Yes |
| HP31 | fHP | GG | GG | 60 | Female | 23.6 | 1.85 | 62 | 6.06 | 32 | No | No | Yes |
| HP32 | fHP | GG | GG | 66 | Male | 21.4 | 3.01 | 72 | 6.5 | 25 | Yes | No | Yes |
| HP33 | fHP | GT | GT+TT | 69 | Female | 39.5 | 2.25 | 79 | 13.24 | 70 | No | No | Yes |
| HP34 | fHP | GG | GG | 60 | Male | 28 | 3.97 | 91 | 8.14 | 31 | No | No | Yes |
| HP35 | fHP | GG | GG | 56 | Female | 25.8 | 1.84 | 57 | 6.9 | 35 | No | No | Yes |
| HP36 | fHP | GG | GG | 61 | Female | 31.6 | 1.87 | 56 | 8.28 | 39 | No | No | No |
| HP37 | fHP | GT | GT+TT | 64 | Female | 26.6 | NA | NA | NA | 34 | No | No | No |
| HP38 | fHP | GG | GG | 44 | Female | 30.1 | 4.03 | 100 | 18.26 | 80 | No | No | No |
| HP39 | fHP | GG | GG | 57 | Female | 25.9 | 1.86 | 57 | 9.16 | 46 | No | No | Yes |

fHP, fibrotic hypersensitivity pneumonitis; IPF, idiopathic pulmonary fibrosis; BMI, body mass index; FVC, forced vital capacity; DLCO, diffusing capacity for carbon monoxide.
COPD, chronic obstructive pulmonary disease. T-carrier group: GG versus GT+TT. NA denotes a missing value.

**Data S1.** Individual genotype and phenotype data. fHP cohort, patients 33-64 of 64.

| Sample | Diagnosis | Genotype | T-carrier group | Age (years) | Sex | BMI ($kg/m^2$) | FVC (L) | FVC (% predicted) | DLCO (mL/min/mmHg) | DLCO (% predicted) | Smoking | COPD | Hypertension |
|---|---|---|---|---|---|---|---|---|---|---|---|---|---|
| HP40 | fHP | GT | GT+TT | 55 | Male | 26.5 | 2.39 | 57 | 10.63 | 42 | Yes | No | Yes |
| HP41 | fHP | GT | GT+TT | 70 | Female | 32.4 | 2.5 | 93 | 10.4 | 56 | No | No | Yes |
| HP42 | fHP | GG | GG | 70 | Male | 35.1 | 3.42 | 80 | 12.74 | 49 | No | No | Yes |
| HP43 | fHP | TT | GT+TT | 84 | Female | 20 | 1.21 | 53 | 7.38 | 43 | No | No | Yes |
| HP44 | fHP | GG | GG | 55 | Female | 39.2 | 2.88 | 79 | 18.52 | 86 | Yes | No | Yes |
| HP45 | fHP | GG | GG | 62 | Male | 34.6 | 3.09 | 71 | 13.24 | 51 | Yes | No | Yes |
| HP46 | fHP | GT | GT+TT | 38 | Female | 24.2 | 1.63 | 57 | 6.27 | 33 | No | No | No |
| HP47 | fHP | GG | GG | 73 | Female | 32 | 2.24 | 81 | 12.45 | 67 | No | No | Yes |
| HP48 | fHP | GT | GT+TT | 35 | Male | 29.4 | 2.53 | 47 | 11.18 | 39 | Yes | No | No |
| HP49 | fHP | GT | GT+TT | 63 | Female | 31.2 | 1.46 | 53 | NA | 34 | Yes | No | Yes |
| HP50 | fHP | GT | GT+TT | 59 | Male | 39 | NA | NA | NA | NA | No | No | Yes |
| HP51 | fHP | GG | GG | 63 | Female | 27.4 | 2.16 | 76 | 8.69 | 49 | No | No | Yes |
| HP52 | fHP | GT | GT+TT | 53 | Female | 31.6 | NA | 57 | NA | 47 | No | No | Yes |
| HP53 | fHP | GG | GG | 61 | Female | 23.3 | NA | 68 | NA | 66 | No | No | No |
| HP54 | fHP | GG | GG | 66 | Male | 25.6 | 4.55 | 112 | 24.4 | 98 | Yes | No | Yes |
| HP55 | fHP | GG | GG | 77 | Male | 30 | 3.06 | 93 | 14.94 | 75 | Yes | No | Yes |
| HP56 | fHP | GG | GG | 58 | Female | 23 | 1.54 | 51 | 4.7 | 39 | No | No | No |
| HP57 | fHP | GG | GG | 27 | Male | 25 | 1.91 | 38 | 12.38 | 40 | No | No | No |
| HP58 | fHP | GT | GT+TT | 66 | Female | 34.8 | 2.16 | 71 | 11.61 | 59 | Yes | No | No |
| HP59 | fHP | GG | GG | 46 | Male | 28.3 | 2.5 | 49 | 11.35 | 37 | Yes | No | Yes |
| HP60 | fHP | GG | GG | 72 | Female | 24.1 | 2.63 | 69 | 9.01 | 50 | No | No | Yes |
| HP61 | fHP | TT | GT+TT | 71 | Female | 37.08 | 2.8 | 108 | 14.5 | 81 | No | No | Yes |
| HP62 | fHP | GT | GT+TT | 74 | Female | 42.1 | 2.04 | 86 | 8.36 | 49 | No | No | Yes |
| HP63 | fHP | GG | GG | 63 | Female | 30.8 | 2.31 | 75 | 10.34 | 53 | No | Yes | Yes |
| HP64 | fHP | GG | GG | 78 | Female | 28.7 | 1.9 | 87 | 6.03 | 37 | No | No | Yes |
| HP65 | fHP | TT | GT+TT | 77 | Female | 33.3 | 2.51 | 109 | 12.4 | 73 | No | No | Yes |
| HP66 | fHP | GG | GG | 46 | Male | 32.7 | 1.42 | 41 | 14.5 | 56 | Yes | No | Yes |
| HP67 | fHP | GG | GG | 64 | Female | 27.9 | 1.85 | 85 | 7.51 | 41 | No | No | Yes |
| HP68 | fHP | GG | GG | 73 | Male | 15.7 | 1.66 | 41 | 5.28 | 21 | No | No | Yes |
| HP69 | fHP | GT | GT+TT | 73 | Male | 32.8 | 3.59 | 97 | 10.48 | 45 | No | No | Yes |
| HP70 | fHP | TT | GT+TT | 67 | Male | 30.6 | NA | 60 | NA | 22 | Yes | No | Yes |
| HP71 | fHP | GT | GT+TT | 74 | Female | 27.2 | 3.4 | 124 | 9.03 | 48 | Yes | No | Yes |

fHP, fibrotic hypersensitivity pneumonitis; IPF, idiopathic pulmonary fibrosis; BMI, body mass index; FVC, forced vital capacity; DLCO, diffusing capacity for carbon monoxide.
COPD, chronic obstructive pulmonary disease. T-carrier group: GG versus GT+TT. NA denotes a missing value.

**Data S1.** Individual genotype and phenotype data. IPF cohort, patients 1-25 of 50.

| Sample | Diagnosis | Genotype | T-carrier group | Age (years) | Sex | BMI ($kg/m^2$) | FVC (L) | FVC (% predicted) | DLCO (mL/min/mmHg) | DLCO (% predicted) | Smoking | COPD | Hypertension |
|---|---|---|---|---|---|---|---|---|---|---|---|---|---|
| IPF1 | IPF | GG | GG | 77 | Male | 27.1 | NA | 56 | NA | 33 | Yes | Yes | Yes |
| IPF2 | IPF | GG | GG | 73 | Male | 26.7 | 4.34 | 109 | 7.24 | 29 | No | No | Yes |
| IPF3 | IPF | GG | GG | 67 | Male | 31.2 | 2.71 | 74 | 9.58 | 42 | No | No | Yes |
| IPF5 | IPF | GT | GT+TT | 77 | Female | 36 | 0.94 | 53 | 4.71 | 34 | No | No | Yes |
| IPF6 | IPF | GG | GG | 75 | Male | 30.2 | 3.73 | 92 | 11.87 | 48 | Yes | No | Yes |
| IPF7 | IPF | GT | GT+TT | 73 | Male | 24.9 | 4.38 | 117 | 12.6 | 54 | No | No | Yes |
| IPF8 | IPF | GG | GG | 73 | Male | 33.5 | 2.27 | 53 | 6.68 | 26 | Yes | No | Yes |
| IPF9 | IPF | GT | GT+TT | 75 | Male | 31 | 3.35 | 64 | 3.8 | 44 | Yes | No | Yes |
| IPF10 | IPF | GT | GT+TT | 68 | Male | 34.3 | 3.25 | 86 | 11.23 | 48 | Yes | Yes | Yes |
| IPF12 | IPF | GT | GT+TT | 74 | Female | 35 | 2.16 | 86 | 11.01 | 62 | Yes | No | Yes |
| IPF13 | IPF | GG | GG | 72 | Male | 30 | 2.29 | 81 | 7.81 | 31 | Yes | No | Yes |
| IPF14 | IPF | GG | GG | 70 | Male | 25.18 | 2.2 | 82 | 3.9 | 40 | Yes | No | No |
| IPF15 | IPF | GT | GT+TT | 75 | Male | 31 | 3.35 | 64 | 3.8 | 44 | Yes | No | Yes |
| IPF16 | IPF | GG | GG | 48 | Male | 39.6 | 2.27 | 52 | 3.57 | 54 | No | No | Yes |
| IPF17 | IPF | GG | GG | 58 | Male | 27.8 | NA | 54 | 4.54 | 29 | No | No | No |
| IPF18 | IPF | GG | GG | 66 | Female | 27.6 | 3.28 | 62 | 9.9 | 24 | Yes | No | Yes |
| IPF19 | IPF | GT | GT+TT | 86 | Male | 29.1 | NA | NA | NA | NA | Yes | No | Yes |
| IPF20 | IPF | GG | GG | 68 | Female | 34.8 | 1.96 | 73 | 5.22 | 29 | No | No | Yes |
| IPF21 | IPF | GG | GG | 70 | Female | 33.6 | 1.95 | 78 | 9.13 | 53 | No | No | Yes |
| IPF22 | IPF | GG | GG | 52 | Male | 23.2 | 1.21 | 28 | 7.14 | 27 | No | No | Yes |
| IPF23 | IPF | GG | GG | 76 | Male | 33.3 | 3.22 | 79 | 10.4 | 44 | No | No | Yes |
| IPF24 | IPF | GT | GT+TT | 42 | Male | 28.7 | 2.45 | 51 | 9.99 | 35 | Yes | No | No |
| IPF25 | IPF | GG | GG | 73 | Male | 19.8 | 4.26 | 109 | 10.06 | 42 | Yes | Yes | Yes |
| IPF26 | IPF | GG | GG | 66 | Male | 19.4 | 2.74 | 74 | 8.48 | 37 | Yes | Yes | Yes |
| IPF27 | IPF | GT | GT+TT | 49 | Female | 29.5 | 2.91 | 93 | 10.12 | 53 | Yes | No | No |

fHP, fibrotic hypersensitivity pneumonitis; IPF, idiopathic pulmonary fibrosis; BMI, body mass index; FVC, forced vital capacity; DLCO, diffusing capacity for carbon monoxide.
COPD, chronic obstructive pulmonary disease. T-carrier group: GG versus GT+TT. NA denotes a missing value.

**Data S1.** Individual genotype and phenotype data. IPF cohort, patients 26-50 of 50.

| Sample | Diagnosis | Genotype | T-carrier group | Age (years) | Sex | BMI ($kg/m^2$) | FVC (L) | FVC (% predicted) | DLCO (mL/min/mmHg) | DLCO (% predicted) | Smoking | COPD | Hypertension |
|---|---|---|---|---|---|---|---|---|---|---|---|---|---|
| IPF28 | IPF | GT | GT+TT | 66 | Male | 30.1 | 2.37 | 62 | 8.33 | 35 | Yes | No | Yes |
| IPF29 | IPF | GT | GT+TT | 79 | Male | 20.1 | NA | 83 | NA | 48 | No | No | No |
| IPF30 | IPF | GT | GT+TT | 77 | Male | 25.4 | 2.76 | 61 | 10.64 | 39 | No | No | Yes |
| IPF31 | IPF | GT | GT+TT | 63 | Male | 32.8 | NA | 67 | NA | 46 | Yes | No | No |
| IPF34 | IPF | TT | GT+TT | 63 | Male | 38.6 | 2.19 | 40 | NA | 41 | Yes | No | Yes |
| IPF36 | IPF | GT | GT+TT | 56 | Male | 31.2 | 2.71 | 74 | 9.58 | 42 | No | No | Yes |
| IPF37 | IPF | GT | GT+TT | 67 | Female | 29.2 | 1.44 | 66 | 6.4 | 36 | No | No | Yes |
| IPF38 | IPF | GG | GG | 64 | Male | 30 | 4.2 | 62 | 11.34 | 44 | No | No | No |
| IPF39 | IPF | GG | GG | 70 | Male | 30 | 3.69 | 70 | 3.84 | 69 | Yes | No | Yes |
| IPF40 | IPF | GT | GT+TT | 64 | Male | 19.2 | 2.48 | 47 | 4.82 | 16 | Yes | No | Yes |
| IPF41 | IPF | GT | GT+TT | 62 | Male | 27.5 | 2.48 | 65 | 8.49 | 36 | Yes | No | No |
| IPF42 | IPF | GG | GG | 70 | Male | 35.1 | 3.3 | 82 | 18.38 | 76 | Yes | No | Yes |
| IPF43 | IPF | GG | GG | 32 | Male | 22.7 | 2.84 | 67 | 6.95 | 22 | Yes | No | Yes |
| IPF44 | IPF | GT | GT+TT | 67 | Female | 34 | 1.39 | 51 | 5.56 | 30 | No | No | Yes |
| IPF45 | IPF | GT | GT+TT | 60 | Female | 25.1 | 1.02 | 47 | 6.24 | 35 | No | No | No |
| IPF46 | IPF | GT | GT+TT | 75 | Male | 27.4 | NA | 95 | NA | NA | Yes | No | Yes |
| IPF47 | IPF | TT | GT+TT | 56 | Male | 26.1 | 2.9 | 62 | 11.25 | 41 | Yes | No | Yes |
| IPF49 | IPF | GT | GT+TT | 85 | Female | 22.6 | 1.75 | 83 | 6.1 | 37 | Yes | Yes | No |
| IPF50 | IPF | GG | GG | 64 | Male | 32.8 | 3.91 | 75 | 10.22 | 33 | No | Yes | Yes |
| IPF51 | IPF | GT | GT+TT | 55 | Male | 28 | 1.8 | 43 | 9.37 | 36 | No | No | Yes |
| IPF52 | IPF | GT | GT+TT | 65 | Male | 20.9 | 2.22 | 55 | 11.2 | 46 | No | No | Yes |
| IPF53 | IPF | GG | GG | 72 | Female | 24.4 | 1.15 | 57 | 7.72 | 51 | No | No | Yes |
| IPF54 | IPF | GG | GG | 76 | Male | 24.6 | 3.26 | 85 | 14.51 | 61 | No | No | Yes |
| IPF55 | IPF | GG | GG | 64 | Male | 33.9 | 2.76 | 61 | 15.6 | 58 | No | No | Yes |
| IPF56 | IPF | GT | GT+TT | 80 | Female | 35.5 | 1.98 | 89 | 7.66 | 47 | No | No | Yes |

fHP, fibrotic hypersensitivity pneumonitis; IPF, idiopathic pulmonary fibrosis; BMI, body mass index; FVC, forced vital capacity; DLCO, diffusing capacity for carbon monoxide.
COPD, chronic obstructive pulmonary disease. T-carrier group: GG versus GT+TT. NA denotes a missing value.

## Supplementary Tables

**Table S1.** Genotype–phenotype comparisons in the combined patient cohort under the dominant model.

| Variable | GG (n = 61) | T carriers (n = 53) | Unadjusted p | Holm-adjusted p |
|---|---|---|---|---|
| Age, years | 65.0 [57.0–70.0] (n=61) | 67.0 [61.0–74.0] (n=53) | 0.094 | 0.935 |
| Male sex, n (%) | 33/61 (54.1%) | 25/53 (47.2%) | 0.573 | 1.000 |
| BMI, kg/m² | 29.0 [24.8–31.9] (n=60) | 30.1 [26.5–34.0] (n=53) | 0.167 | 1.000 |
| FVC, L | 2.6 [1.9–3.3] (n=58) | 2.2 [1.8–2.8] (n=45) | 0.130 | 1.000 |
| FVC, % predicted | 72.0 [56.0–82.0] (n=61) | 64.0 [53.0–86.0] (n=50) | 0.838 | 1.000 |
| DLCO, mL/min/mmHg | 8.6 [6.5–11.3] (n=58) | 9.0 [6.1–11.2] (n=43) | 0.866 | 1.000 |
| DLCO, % predicted | 40.5 [32.5–55.0] (n=60) | 42.5 [35.0–49.0] (n=50) | 0.864 | 1.000 |
| Smoking, n (%) | 20/61 (32.8%) | 21/53 (39.6%) | 0.558 | 1.000 |
| COPD, n (%) | 5/60 (8.3%) | 2/53 (3.8%) | 0.445 | 1.000 |
| Hypertension, n (%) | 49/60 (81.7%) | 38/53 (71.7%) | 0.264 | 1.000 |

Data are median [Q1–Q3] (available n) for continuous variables and n/N (%) for categorical variables. Continuous variables were compared using the two-sided Mann–Whitney U test, and categorical variables using the two-sided Fisher exact test. Holm adjustment was applied across the 10 variables within this analysis.

**Table S2.** Genotype–phenotype comparisons within the fHP group under the dominant model.

| Variable | GG (n = 37) | T carriers (n = 27) | Unadjusted p | Holm-adjusted p |
|---|---|---|---|---|
| Age, years | 62.0 [55.0–68.0] (n=37) | 69.0 [59.0–72.0] (n=27) | 0.054 | 0.526 |
| Male sex, n (%) | 13/37 (35.1%) | 7/27 (25.9%) | 0.586 | 1.000 |
| BMI, kg/m² | 28.5 [24.8–31.6] (n=36) | 31.2 [26.6–35.5] (n=27) | 0.053 | 0.526 |
| FVC, L | 2.4 [1.9–3.1] (n=36) | 2.2 [1.8–2.6] (n=23) | 0.555 | 1.000 |
| FVC, % predicted | 71.0 [51.0–85.0] (n=37) | 63.0 [56.0–89.0] (n=25) | 0.651 | 1.000 |
| DLCO, mL/min/mmHg | 8.7 [6.1–12.4] (n=35) | 9.3 [6.3–11.4] (n=22) | 0.974 | 1.000 |
| DLCO, % predicted | 40.5 [34.0–65.0] (n=36) | 44.0 [34.0–59.0] (n=26) | 0.966 | 1.000 |
| Smoking, n (%) | 9/37 (24.3%) | 6/27 (22.2%) | 1.000 | 1.000 |
| COPD, n (%) | 1/36 (2.8%) | 0/27 (0.0%) | 1.000 | 1.000 |
| Hypertension, n (%) | 28/36 (77.8%) | 19/27 (70.4%) | 0.566 | 1.000 |

Data are median [Q1–Q3] (available n) for continuous variables and n/N (%) for categorical variables. Continuous variables were compared using the two-sided Mann–Whitney U test, and categorical variables using the two-sided Fisher exact test. Holm adjustment was applied across the 10 variables within this analysis.

**Table S3.** Genotype–phenotype comparisons within the IPF group under the dominant model.

| Variable | GG (n = 24) | T carriers (n = 26) | Unadjusted p | Holm-adjusted p |
|---|---|---|---|---|
| Age, years | 70.0 [64.0–73.0] (n=24) | 67.0 [62.0–75.0] (n=26) | 0.861 | 1.000 |
| Male sex, n (%) | 20/24 (83.3%) | 18/26 (69.2%) | 0.327 | 1.000 |
| BMI, kg/m² | 30.0 [24.9–33.4] (n=24) | 29.1 [25.4–32.8] (n=26) | 0.969 | 1.000 |
| FVC, L | 2.8 [2.3–3.7] (n=22) | 2.4 [1.8–2.9] (n=22) | 0.078 | 0.783 |
| FVC, % predicted | 73.5 [59.0–81.5] (n=24) | 64.0 [53.0–83.0] (n=25) | 0.477 | 1.000 |
| DLCO, mL/min/mmHg | 8.5 [6.7–10.4] (n=23) | 8.5 [6.1–10.6] (n=21) | 0.778 | 1.000 |
| DLCO, % predicted | 41.0 [29.0–52.0] (n=24) | 41.0 [35.5–46.5] (n=24) | 0.703 | 1.000 |
| Smoking, n (%) | 11/24 (45.8%) | 15/26 (57.7%) | 0.572 | 1.000 |
| COPD, n (%) | 4/24 (16.7%) | 2/26 (7.7%) | 0.409 | 1.000 |
| Hypertension, n (%) | 21/24 (87.5%) | 19/26 (73.1%) | 0.294 | 1.000 |

Data are median [Q1–Q3] (available n) for continuous variables and n/N (%) for categorical variables. Continuous variables were compared using the two-sided Mann–Whitney U test, and categorical variables using the two-sided Fisher exact test. Holm adjustment was applied across the 10 variables within this analysis.

**Table S4.** PCR–RFLP genotyping quality-control summary.

| Quality-control metric | Result |
|---|---|
| Total samples | 114 |
| fHP samples | 64 |
| IPF samples | 50 |
| GG genotypes | 61 |
| GT genotypes | 44 |
| TT genotypes | 9 |
| T-allele carriers | 53 |
| Genotype call rate | 100% |
| Failed genotype calls | 0 |
| Repeated genotype assays | 0 |
| Sanger-validated samples | 3 (one GG, one GT, one TT) |
| Sanger concordance | 3/3 (100%) |

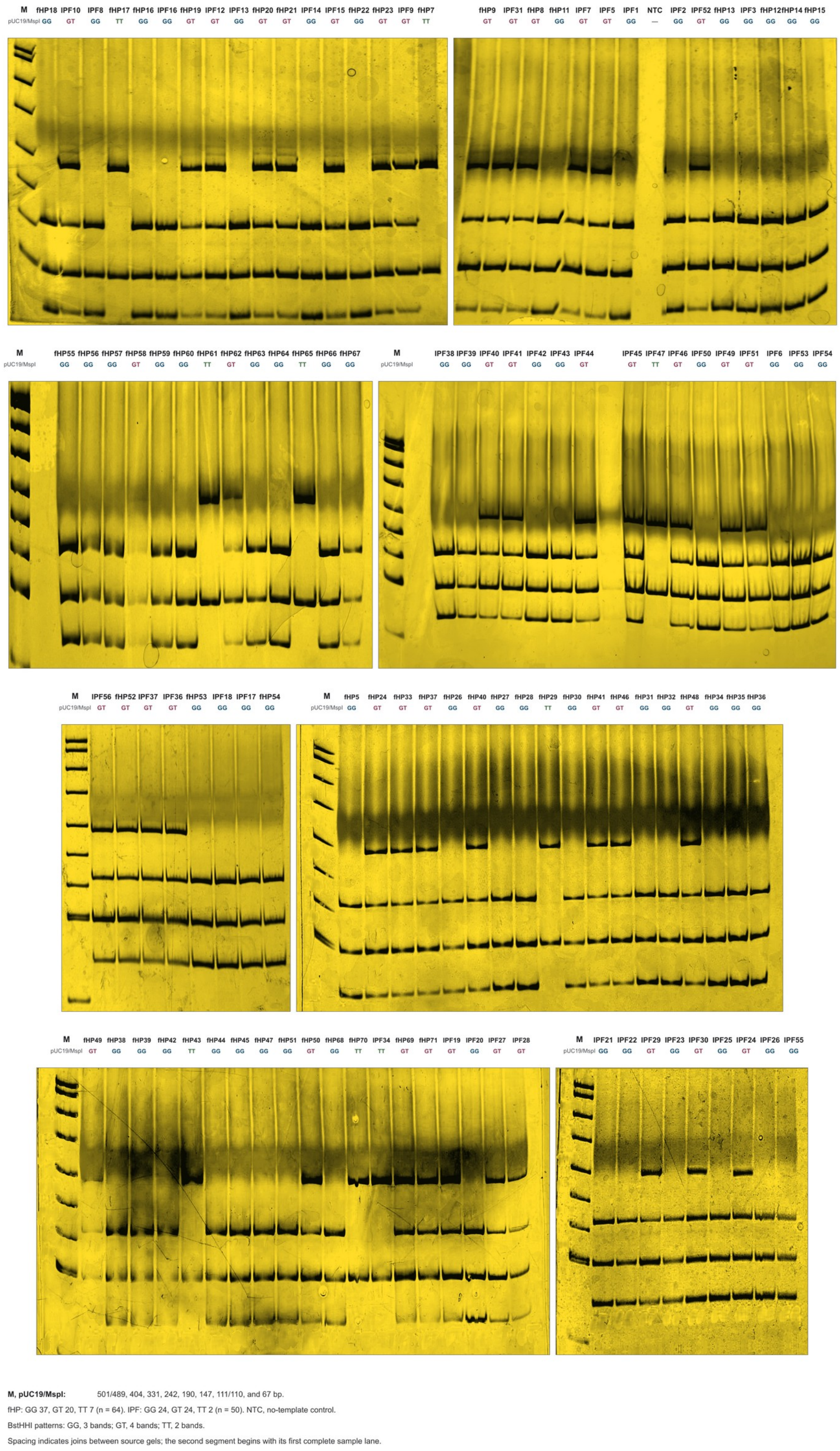


**Figure S1.** Complete PCR–RFLP gel documentation for all 114 patient samples. Each lane is labelled with the anonymized sample code and assigned genotype. M, pUC19/MspI DNA size marker (501/489, 404, 331, 242, 190, 147, 111/110, and 67 bp); NTC, no-template control. Visible BstHHI patterns were three bands for GG, four for GT, and two for TT. The non-polymorphic restriction site served as an internal digestion control. White spacing indicates joins between source gel images. Sanger confirmation of representative GG, GT, and TT calls is shown in main Figure 1.

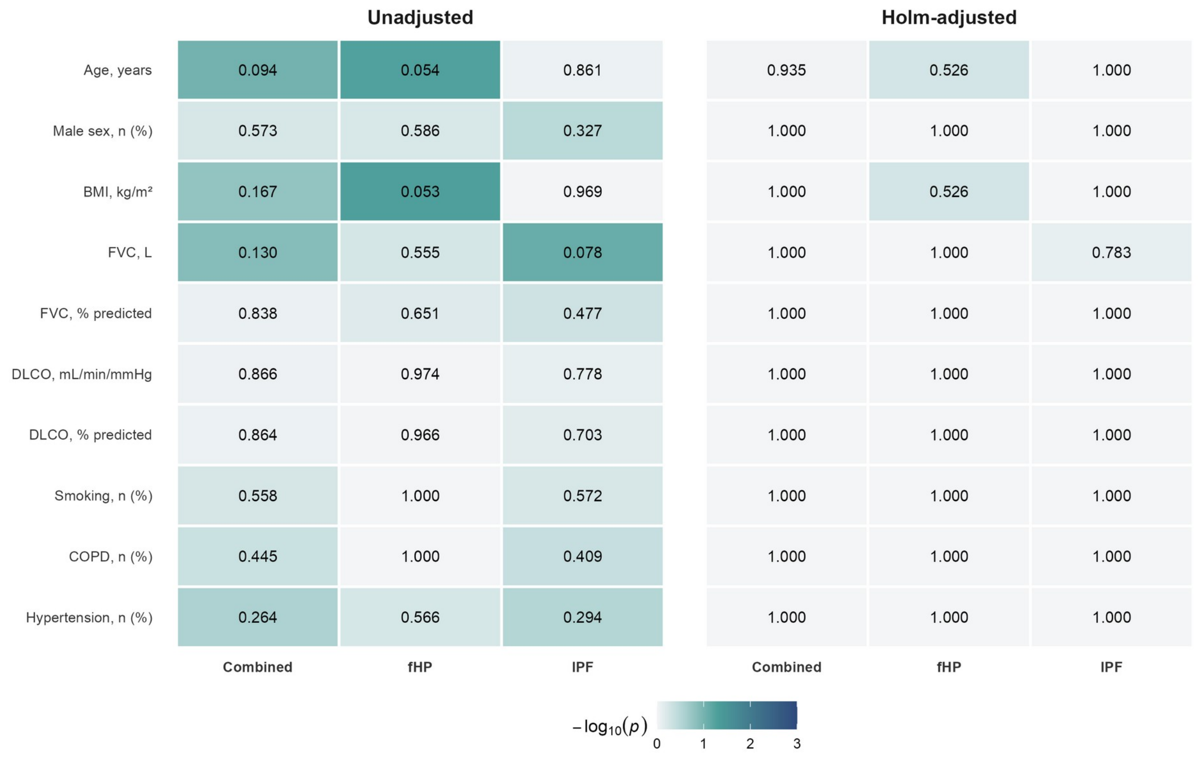


**Figure S2.** Unadjusted and Holm-adjusted p values for dominant-model genotype–phenotype comparisons in the combined cohort and separately in the fHP and IPF groups. Mann–Whitney U tests were used for continuous variables and Fisher exact tests for categorical variables. Values are rounded to three decimal places.

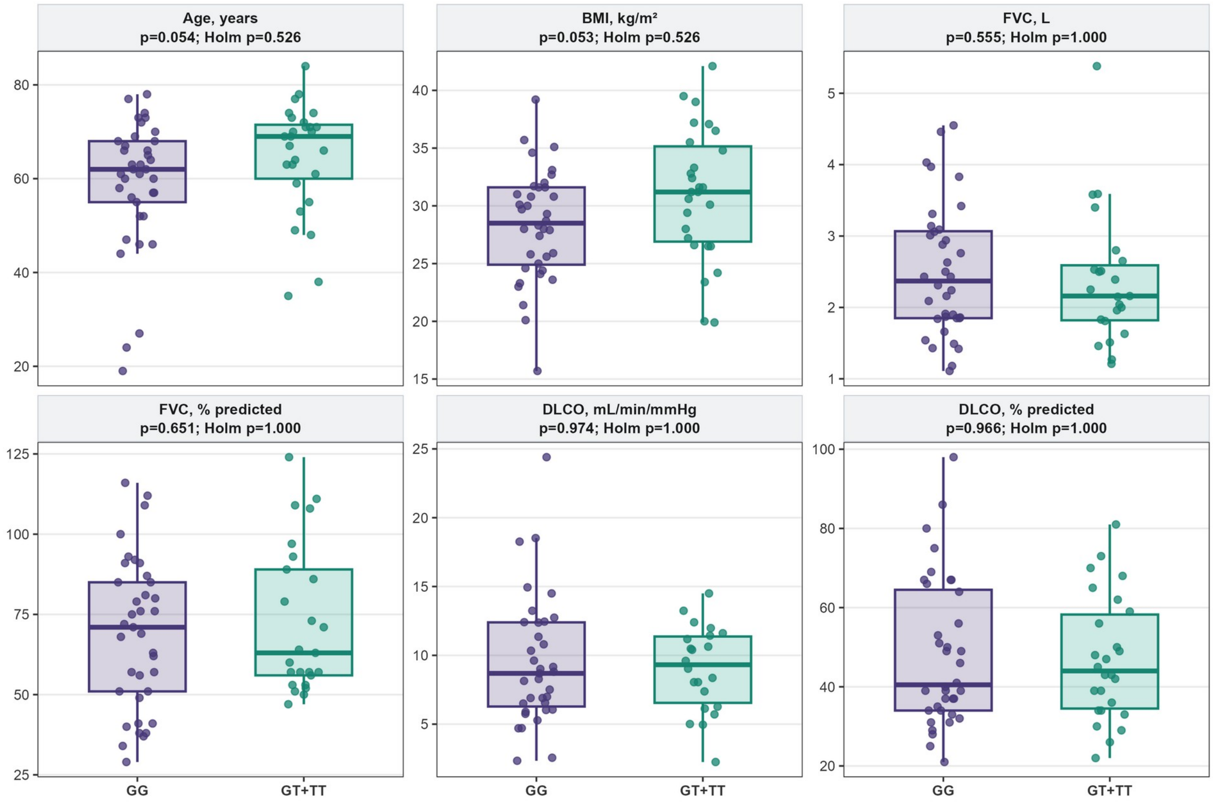


**Figure S3.** Continuous clinical variables according to MUC5B rs35 705 950 T-carrier status in the fHP group. Points show individual observations; boxes show medians and interquartile ranges, and whiskers extend to 1.5 times the interquartile range. Panel titles report unadjusted and Holm-adjusted p values.

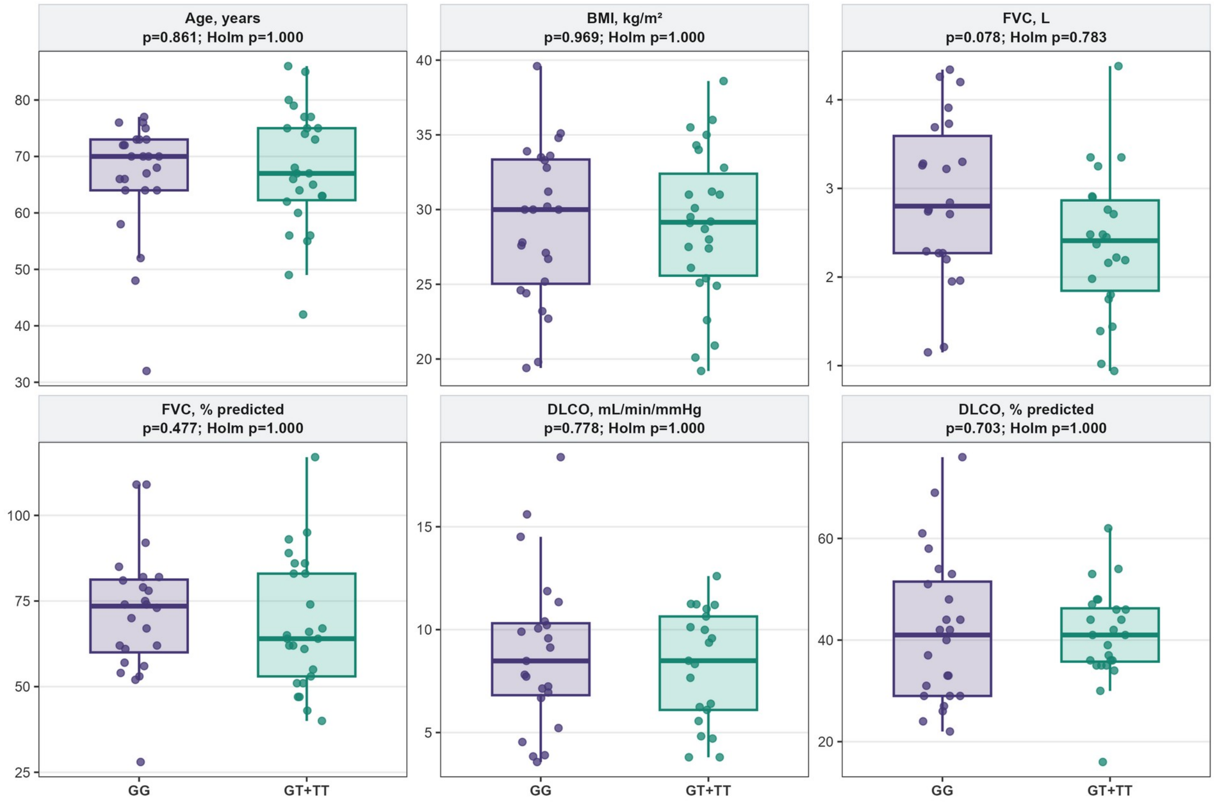


**Figure S4.** Continuous clinical variables according to MUC5B rs35 705 950 T-carrier status in the IPF group. Points show individual observations; boxes show medians and interquartile ranges, and whiskers extend to 1.5 times the interquartile range. Panel titles report unadjusted and Holm-adjusted p values.

**Table S5.** Association of *MUC5B* rs35705950 with fHP in patients with an identified antigen and a non-UIP pattern.

| Model | fHP subgroup n/N (%) | Reference n/N (%) | OR (95% CI) | Unadjusted p | Holm-adjusted p |
|---|---|---|---|---|---|
| Dominant GT + TT vs GG | 14/39 (35.9) | 16,871/89,261 (18.9) | 2.40 (1.15-4.80) | 0.0123 | 0.0123 |
| Allelic T vs G | 17/78 (21.8) | 17,780/178,522 (9.96) | 2.52 (1.38-4.37) | 0.00181 | 0.00361 |

In the dominant model, n/N denotes T-allele carriers/individuals; in the allelic model, it denotes T alleles/total alleles. Both models use the same patients and variant.

A sensitivity analysis was restricted to 39 of the 64 patients with fibrotic hypersensitivity pneumonitis (fHP) who had both an identified inciting antigen and a non-UIP pattern on HRCT. Subgroup membership was based on the available clinical information; genotypes were retrieved from the study dataset. The subgroup comprised 25 GG, 11 GT, and 3 TT genotypes. The 18 patients with a UIP-like pattern and seven with unavailable UIP-like status were not included in this sensitivity analysis.

The same external Russian reference dataset was used (n = 89,261; 72,390 GG, 15,962 GT, and 909 TT). Two-sided Fisher exact tests evaluated the dominant model (GT + TT versus GG) and the allelic model (T versus G). Odds ratios (ORs) were conditional maximum-likelihood estimates with exact conditional 95% confidence intervals (CIs), calculated using stats::fisher.test in R 4.4.2. Holm adjustment was applied across the two tests of this supplementary analysis, separately from the original analysis families. ORs were not adjusted for covariates because only aggregate reference data were available; CIs were not adjusted for multiplicity.

The association persisted under both models after Holm adjustment. This analysis supports persistence of the association in the specified clinical subgroup. Because the subgroup is nested within the original cohort, it is a sensitivity analysis rather than an independent replication; it does not establish the accuracy of individual diagnoses.

Included study sample identifiers: HP5, HP7, HP8, HP9, HP11, HP15, HP16, HP18, HP19, HP20, HP21, HP22, HP23, HP31, HP34, HP36, HP39, HP40, HP41, HP42, HP43, HP44, HP45, HP47, HP48, HP52, HP53, HP54, HP55, HP56, HP57, HP59, HP60, HP61, HP62, HP63, HP64, HP66, HP68.
